# Corvad as a bridge to LVAD implantation in end-stage heart disease complicated by cardiogenic shock: a three-case series

**DOI:** 10.3389/fcvm.2026.1816772

**Published:** 2026-05-25

**Authors:** Jianhao Hu, Wei Li, Chengxin Zhang, Yuyong Liu

**Affiliations:** Department of Cardiovascular Surgery, First Affiliated Hospital of Anhui Medical University, Hefei, China

**Keywords:** cardiogenic shock, CorVad, end-stage heart disease, LVAD, PVAD

## Abstract

**Objective:**

To evaluate the safety, hemodynamic efficacy, and short-term outcomes of CorVad mechanical circulatory support as a bridge to left ventricular assist device (LVAD) therapy for cardiogenic shock and the changes in related hemolysis indicators.

**Methods:**

We retrospectively analyzed three patients with Society for Cardiovascular Angiography and Interventions (SCAI) stage D Cardiogenic shock (CS) admitted between November 2024 and May 2025 to the First Affiliated Hospital of Anhui Medical University. All received CorVad support and were bridged to LVAD. Hemodynamic parameters, short-term prognosis, and dynamic changes in hemoglobin (Hb), hematocrit (Hct) and Indirect bilirubin within 24 h post-implantation were assessed.

**Results:**

CorVad support led to rapid improvements in mean arterial pressure, cardiac output, and cardiac index, accompanied by a marked reduction in lactate. All three patients successfully underwent elective LVAD implantation without procedural complications or in-hospital mortality. All patients remained alive and ambulatory at 30-day and 3-month follow-up. Within 24 h post-implantation, Hb, Hct declined modestly and indirect bilirubin levels have slightly increased, suggesting mild hemolysis or transient hemodilution, without severe hemolysis-related complications.

**Conclusion:**

CorVad provides effective and timely hemodynamic rescue in patients with CS, enabling safe transition to durable LVAD implantation, with favorable short-term prognosis. Serial monitoring of hemolysis-related indicators is required postoperatively to identify and manage potential hemolysis risks at an early stage.

## 1 Introduction

**Figure 1:**
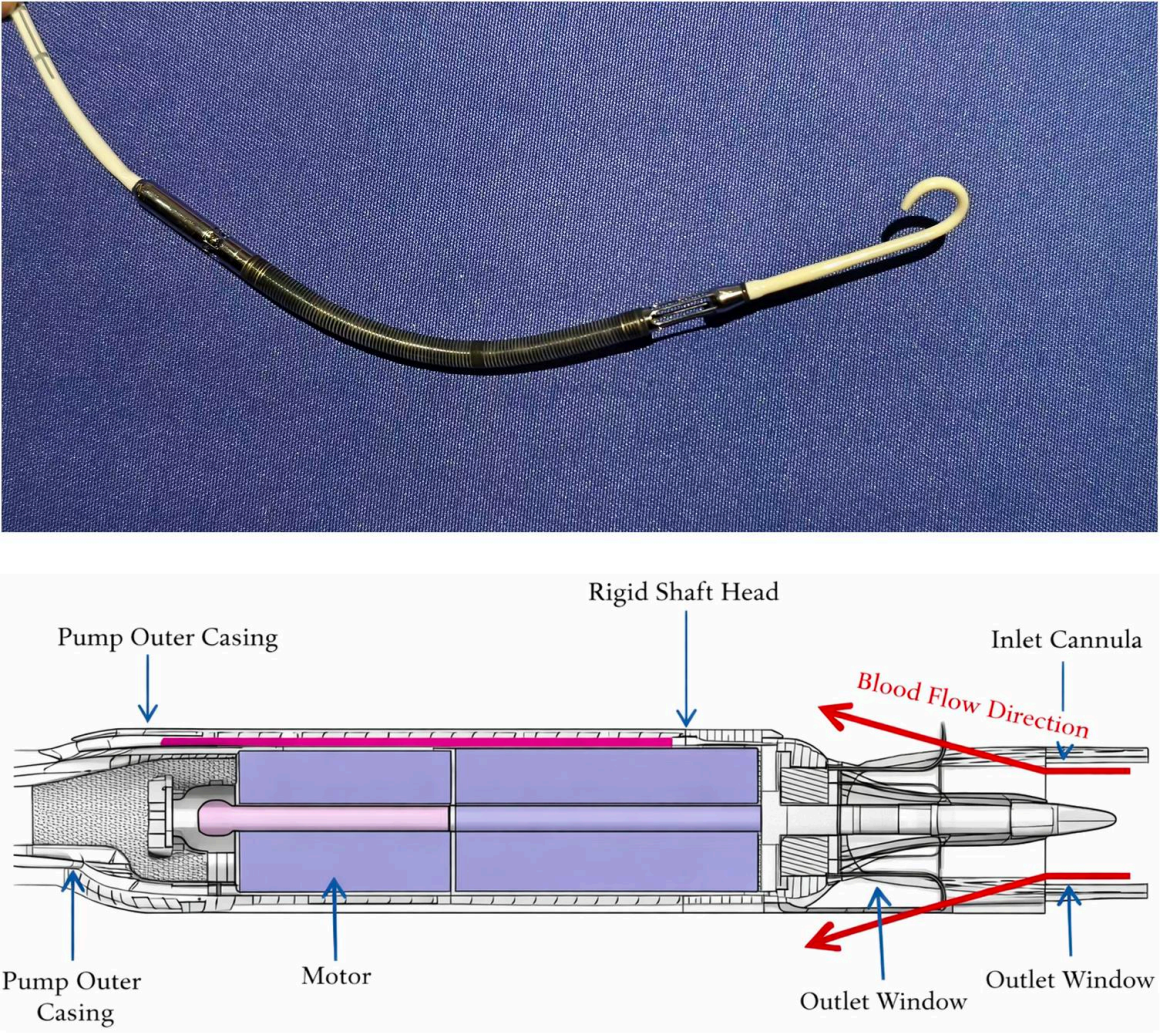
The pump head and cross-sectional view of the CorVad device.

Cardiogenic shock (CS) remains a life-threatening complication of end-stage heart failure, associated with mortality exceeding 50% despite contemporary pharmacological and mechanical interventions (1, 2). For patients requiring durable LVAD implantation, preoperative refractory CS creates a high-risk emergency scenario that demands rapid, effective hemodynamic stabilization. Conventional strategies including intra-aortic balloon pump (IABP) and veno-arterial extracorporeal membrane oxygenation (VA-ECMO) offer suboptimal left ventricular unloading and may impede subsequent LVAD cannulation or increase bleeding and thromboembolic risks. CorVad (Figure 1) is a novel percutaneous ventricular assist device (pVAD) designed to aspirate blood directly from the left ventricle into the ascending aorta, thereby achieving simultaneous ventricular decompression and systemic perfusion augmentation. However, clinical experience with CorVad as a deliberate bridge to elective LVAD implantation remains scarce. This case series presents three patients with end-stage heart disease and SCAI stage D CS who underwent successful CorVad-supported stabilization followed by planned, elective durable LVAD implantation. We report their hemodynamic response, procedural safety, and short-term clinical outcomes to inform the evolving role of CorVad in advanced heart failure management.

## 2 Methods

### 2.1 Research design and subjects

This study is a single-center, retrospective case series conducted at the First Affiliated Hospital of Anhui Medical University. Three patients with end-stage heart failure and CS between November 2024 and May 2025 were included. All patients received CorVad percutaneous left ventricular assist device support and were bridged to LVAD after hemodynamic stabilization.

All patients met standardized diagnostic criteria for CS: (i) persistent hypotension (systolic blood pressure < 90 mmHg or dependence on vasoactive agents after adequate volume resuscitation), (iI) cardiac index < 2.2 L min⁻¹ m⁻², and evidence of tissue hypoperfusion (e.g., elevated lactate, oliguria). All patients had severe left ventricular dysfunction and deemed eligible for long-term mechanical circulatory support per multidisciplinary heart failure and mechanical circulatory support team consensus.

This study was approved by the Ethics Committee of the First Affiliated Hospital of Anhui Medical University. Written informed consent was obtained from all patients or their legally authorized representatives.

### 2.2 Corvad device and bridging protocol

CorVad is a percutaneously inserted, magnetically levitated micro-axial flow pump system that aspirates blood from the left ventricle into the ascending aorta, achieving left ventricular unloading and improving systemic perfusion.

This study used a short-term mechanical circulatory support bridging strategy: CorVad was implanted in the acute phase to rapidly stabilize hemodynamics, allowing time for comprehensive cardiac assessment and subsequent elective long-term LVAD implantation.

### 2.3 Inclusion and exclusion criteria

Inclusion Criteria

(1) Age ≥ 18 years;
(2) Confirmed diagnosis of CS;
(3) End-stage or progressive heart failure with LVEF < 35%;
(4) Persistent hemodynamic instability despite maximal medical therapy and/or prior mechanical support (IABP, ECMO);
(5) Clinical indication and candidacy for durable LVAD implantation.

Exclusion Criteria

(1) Non-cardiogenic shock;
(2) Severe irreversible right heart failure;
(3) Severe aortic valve disease or mechanical valve prosthesis;
(4) Echocardiographically confirmed left ventricular thrombus;
(5) Severe peripheral vascular disease precluding cannulation;
(6) Contraindication to anticoagulation or active bleeding.

### 2.4 Corvad implantation procedure

Under general anesthesia, arterial access was obtained via the femoral artery. Under real-time fluoroscopic or ultrasound guidance, a guidewire was advanced across the aortic valve into the left ventricular cavity. The CorVad catheter pump system was inserted along the guidewire into the left ventricular cavity, ensuring the pump head positioned appropriately below the aortic valve. Upon deployment, the device was activated incrementally based on hemodynamics to increase cardiac output and reduce vasoactive infusions, achieving sufficient left ventricular unloading.

### 2.5 Perioperative management and bridging process

(1) Anticoagulation strategy

Intravenous heparin was infused postoperatively to maintain activated partial thromboplastin time (APTT) within a therapeutic range of 40–60 s.

(1) Circulation support management

The following parameters were monitored continuously to guide support:

- Mean arterial pressure (MAP)
- Cardiac index (CI)
- Blood lactate
- Timing of bridging to LVAD

Bridging to durable LVAD was pursued only after fulfillment of all following criteria:

(1) Hemodynamic stability (CI ≥ 2.2 L min⁻¹ m⁻²)
(2) Lactate level decreased to ≤ 2.0 mmol/L and remained stable
(3) Improvement of end-organ function
(4) Persistent irreversible cardiac dysfunction

Elective LVAD implantation was then performed to transition from short-term to long-term mechanical circulatory support.

### 2.6 Outcome measures

(1) Hemodynamic Indicators: cardiac output (CO), CI
(2) Cardiac Function Indicators: LVEF, LVEDD
(3) Perfusion Indicators: Lactate, urine output
(4) Safety Indicators: Hemolysis, bleeding, vascular complications, arrhythmia
(5) Clinical Outcomes: CorVad success; LVAD bridging success; in-hospital mortality; 30-day survival; 3-month survival

### 2.7 Statistical methods

Due to the small sample size (*n* = 3), no formal statistical analyses were performed. Data are presented using descriptive methods to characterize the clinical course of each patient. Continuous variables are reported as raw values or ranges, while categorical variables are expressed as absolute frequencies.

## 3 Result

This study enrolled three male patients who received CorVad mechanical circulatory support and were bridged to LVAD for CS. All three patients were in New York Heart Association (NYHA) functional class IV and SCAI stage D CS. Baseline characteristics, changes during CorVad support, and short-term prognosis are summarized below.

### 3.1 Baseline clinical characteristics

All three patients had valvular regurgitation as the primary cardiac disease. Cases 1 and 3 had severe mitral and tricuspid regurgitation; Case 2 had patent foramen ovale with severe mitral regurgitation and mild-to-moderate aortic regurgitation. Case 3 had hypertension and diabetes; Case 2 had a history of cerebral infarction; and no evidence of coronary artery disease in any patient.

Laboratory tests showed abnormal myocardial injury markers, elevated lactate, and leukocytosis. Red blood cell counts, Hb, and Hct were within normal ranges. Liver function was impaired with elevated total bilirubin. Detailed data are shown in Table 1.

**Table 1:** Baseline characteristics of patients.

Item	Unit	Case 1	Case 2	Case 3
Age	Years	34	77	43
Sex	-	Male	Male	Male
Body Mass Index (BMI)	kg/m²	25.0	18.3	34.0
Body Surface Area (BSA)	m²	2.17	1.57	2.50
Past Medical History				
Hypertension	-	No	No	Yes
Diabetes Mellitus	-	No	No	Yes
Coronary Heart Disease	-	No	No	No
Cerebral Infarction	-	No	Yes	No
Cardiac Function Class	-	IV	IV	IV
SCAI Classification	-	Stage D	Stage D	Stage D
B-type Natriuretic Peptide (BNP)	pg/mL	2,728.73	2,962.80	781.56
Lactic Acid	mmol/L	3.3	2.3	3.2
White Blood Cell Count	× 10⁹/L	17.86	11.07	11.65
Red Blood Cell Count	× 10¹²/L	4.19	4.82	4.20
Hemoglobin (Hb)	g/L	126	142	132
Hematocrit (HCT)	%	38.7	43.9	42.3
Platelet Count	× 10⁹/L	251	132	166
Alanine Aminotransferase (ALT)	U/L	76.6	23.9	201.6
Aspartate Aminotransferase (AST)	U/L	37.2	92.6	82.6
Total Bilirubin (TBil)	μmol/L	72.44	43.51	56.71
Urine White Blood Cells	-	1+	1+	-
Urine Red Blood Cells	-	2+	3+	-
Urine Protein	-	-	3+	-

Baseline characteristics were obtained at hospital admission.

### 3.2 Hemodynamic and functional response to CorVad support

After CorVad implantation, all patients showed significant improvements in their cardiac output, cardiac index, MAP, and lactate clearance rate. Cardiac structure and function improved mildly: LVEF increased slightly, and LVEDD decreased in all cases.

No vascular complications occurred. Preexisting arrhythmias remained unchanged, and no new arrhythmias developed. Detailed data are shown in Table 2.

**Table 2:** Changes in related indicators before and after CorVad device implantation.

Item	Unit	Case 1	Case 2	Case 3
Support Duration	d	7	7	7
Mean Arterial Pressure (MAP)	mmHg			
Before implantation	mmHg	65	62	63
After implantation	mmHg	76	74	71
Cardiac Output (CO)	L/min			
Before implantation	L/min	2.5	2.2	2.7
After implantation	L/min	4.4	3.6	4.8
Cardiac Index (CI)	L/(min·m²)			
Before implantation	L/(min·m²)	1.80	1.70	1.85
After implantation	L/(min·m²)	2.80	2.65	3.10
Lactic Acid	mmol/L			
Before implantation	mmol/L	3.3	3.4	3.2
After implantation	mmol/L	1.9	1.6	1.8
Ejection Fraction (EF)	%			
Before implantation	%	28	18	30
After implantation	%	29	20	32
Left Ventricular End-Diastolic Diameter (LVEDD)	cm			
Before implantation	cm	7.1	8.5	8.8
After implantation	cm	6.82	7.50	8.30
Tricuspid Annular Plane Systolic Excursion (TAPSE)	cm			
Before implantation	cm	1.37	1.50	1.33
After implantation	cm	1.45	1.62	1.70
Inner Diameter of Proximal Inferior Vena Cava	cm			
Before implantation	cm	2.29	1.80	3.29
After implantation	cm	1.36	1.40	2.60
Serum Creatinine (Scr)	μmol/L			
Before implantation	μmol/L	286(CRRT)	352	258(CRRT)
After implantation	μmolL	221	295	209
Blood Urea Nitrogen (BUN)	mmol/L			
Before implantation	mmol/L	18.2(CRRT)	24.7	16.5(CRRT)
After implantation	mmol/L	12.1	17.8	10.5
Vascular Complications	-			
Before implantation	-	No	No	No
After implantation	-	No	No	No
Arrhythmia	-			
Before implantation	-	No	Atrial Premature Beats	Ventricular Premature Beats
After implantation	-	No	Atrial Premature Beats	Ventricular Premature Beats

Before: immediately before CorVad implantation; After: 24 h after CorVad implantation.

### 3.3 Hematologic monitoring for hemolysis after CorVad implantation

**Figure 2:**
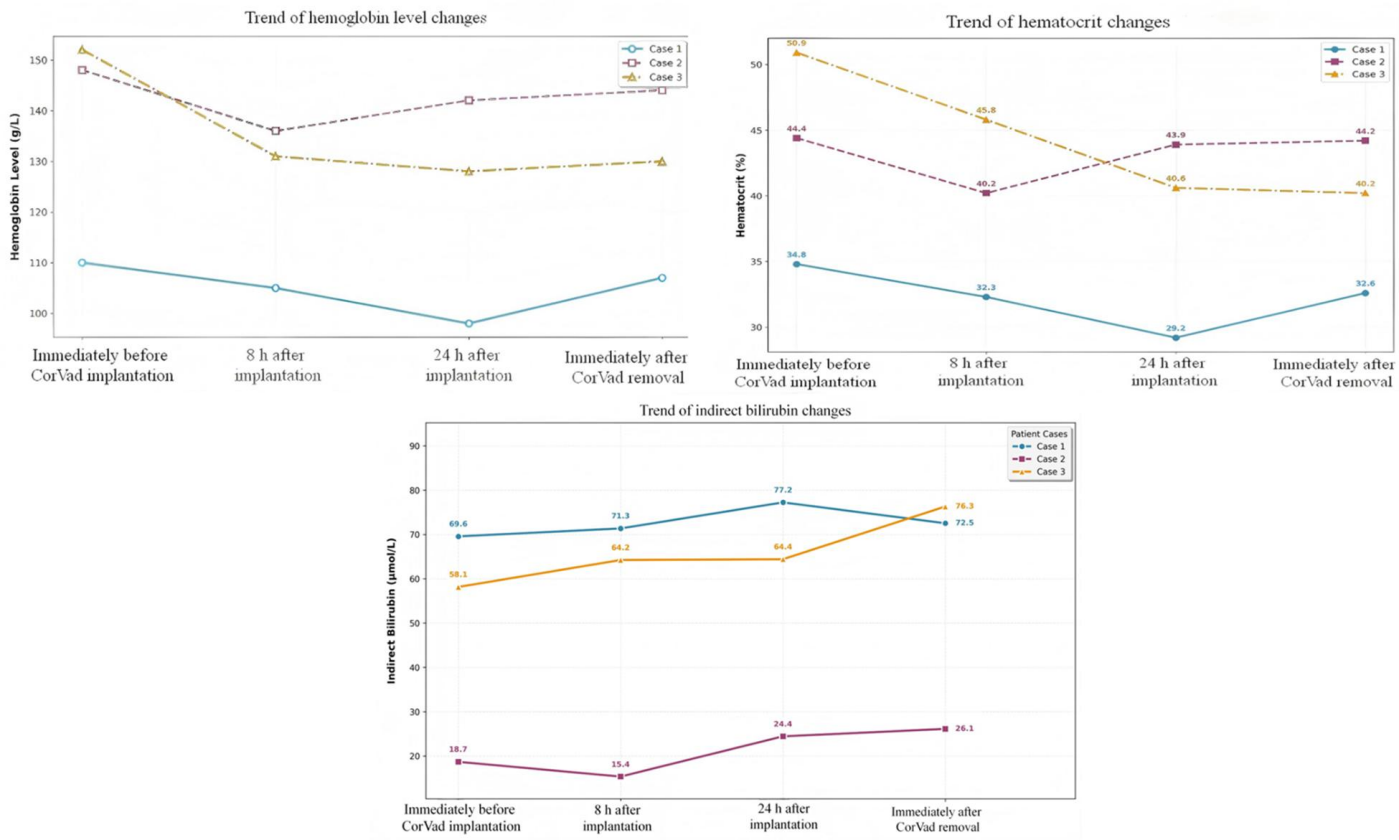
Indirect hemolysis indicators during CorVad implantation and removal.

To prospectively evaluate the effect of CorVad on hematological parameters, Hb, Hct and indirect bilirubin were measured at 8 h and 24 h post-implantation to assess potential hemolysis. Detailed changes are shown in Figure 2.

### 3.4 Short-term prognosis

All three patients successfully completed CorVad support and were electively bridged to LVAD. No in-hospital mortality occurred. At 30-day follow-up, all patients were alive, ambulatory, and discharged home, with further improvements in hemodynamics and cardiac function. Detailed data are shown in Table 3.

**Table 3:** Short-term prognosis and follow-up Status.

Item	Unit	Case 1	Case 2	Case 3
Ejection Fraction (EF)	%	31	36	27
Left Ventricular End-Diastolic Diameter (LVEDD)	cm	6.52	6.40	7.80
Successful CorVad Support	-	Yes	Yes	Yes
Successful LVAD Bridging	-	Yes	Yes	Yes
In-hospital Mortality	-	No	No	No
30-day Survival	-	Alive	Alive	Alive
3-month Survival	-	Alive	Alive	Alive

Echocardiographic data were obtained at 3 months after LVAD implantation.

## 4 Discussion

The pathophysiology of cardiogenic shock centers on acute, severe impairment of cardiac output, precipitating global tissue hypoperfusion, cellular dysoxia, and progressive multiorgan dysfunction. In this context, timely mechanical circulatory support is not merely adjunctive—it is a prerequisite for organ recovery and eligibility assessment for long-term circulatory support (3, 4).

In this case series, CorVad demonstrated rapid, reliable, and titratable hemodynamic rescue in three high-acuity patients with SCAI stage D CS and end-stage heart failure. Shortly after initiation of CorVad support, significant improvements were observed in cardiac output, cardiac index, and mean arterial pressure, accompanied by enhanced lactate clearance. These findings confirm that CorVad can effectively reverse tissue hypoperfusion and restore end-organ function, supporting its role as a reliable percutaneous bridge-to-LVAD strategy in critically ill patients.

Compared with VA-ECMO, CorVad provides selective left ventricular unloading without augmenting afterload or inducing retrograde aortic flow, a key advantage that mitigates myocardial stress and enables a more stable transition to long-term mechanical support (5). In contrast to IABP, which has not demonstrated consistent survival benefits in patients with CS (6, 7), CorVad delivers active, flow-dependent circulatory support with immediate hemodynamic effect, making it uniquely suited for hemodynamically unstable patients requiring time for comprehensive LVAD candidacy assessment.

Device-related hemolysis remains a critical safety consideration in mechanical circulatory support. Its pathogenesis involves shear-mediated erythrocyte fragmentation at high-velocity interfaces—particularly within the pump head and narrow cannula lumens. Compared with other mechanical circulatory support devices, the hemolysis risk of CorVad appears controllable. Previous studies reported a 5%–15% incidence of hemolysis with short-term axial or centrifugal pumps, with severe hemolysis requiring transfusion or intervention accounting for less than 3% (8). In our series, serial monitoring, including Hb, Hct and indirect bilirubin, confirmed absence of clinically significant intravascular hemolysis. Although mild decreases in hemoglobin and hematocrit were observed within 24 h, no clinically significant hemolysis was detected. This safety profile is consistent with reports of contemporary percutaneous microaxial-flow pumps, supporting the perioperative feasibility of CorVad in this high-risk population.

All three patients successfully transitioned from CorVad to LVAD implantation, with zero in-hospital mortality. No serious adverse events occurred in the three patients during the CorVad support period and perioperative period. There was no obvious hemolysis, no severe bleeding, no vascular complications, no thromboembolism, no new arrhythmia, and no device-related complications. At 30-day and 3-month follow-up, all remained alive, ambulatory, and demonstrated progressive improvements in hemodynamic and cardiac functional parameters. These outcomes substantiate the clinical feasibility and short-term efficacy of the “CorVad-LVAD” bridging strategy in patients with end-stage heart failure complicated by CS. Importantly, unlike VA-ECMO–based bridging, CorVad support was not associated with severe vascular complications, infection, or bleeding, which may be attributed to its more physiological support pattern and smaller blood-contacting surface area. CorVad therefore a pragmatic, physiology-aligned bridging modality for patients in whom rapid stabilization must coexist with meticulous pre-LVAD optimization.

### 4.1 Study limitations

This study has several limitations. First, it is a single-center, retrospective case series comprising only three patients; therefore, findings can only be used as a reference. Second, longitudinal serial hemodynamic data were not available. Third, although Hb, Hct and indirect bilirubin were measured prospectively in all cases, other markers of subclinical hemolysis were not systematically collected Fourth, follow-up duration was relatively short (3 months). Prospective, multicenter studies with standardized endpoints and extended follow-up are warranted to validate these preliminary observations and define CorVad's role within evolving mechanical circulatory support algorithms.

## 5 Conclusion

In conclusion, CorVad mechanical circulatory support can provide rapid, titratable, and physiologically appropriate hemodynamic rescue in patients with end-stage heart failure and refractory CS. It enables safe, elective transition to durable LVAD therapy with excellent early survival and functional recovery. Clinically, it is necessary to strengthen the monitoring of hemolysis-related indicators and intervene early to avoid serious complications. This case series offers preliminary clinical evidence supporting CorVad as a viable bridge to help patients with CS survive the critical period and smoothly transition to LVAD. Larger prospective studies are essential to confirm long-term safety, durability, and comparative effectiveness against established bridging modalities.

## Data Availability

The original contributions presented in the study are included in the article/Supplementary Material, further inquiries can be directed to the corresponding authors.
